# Advancing Near-Term Water Quality Forecasting with Explainable Machine Learning and Probabilistic Uncertainty Quantification

**DOI:** 10.1021/acsestwater.6c00757

**Published:** 2026-09-14

**Authors:** Rohit Shukla, Cayelan C. Carey, Adrienne Breef-Pilz, R. Quinn Thomas

**Affiliations:** † Department of Biological Sciences, 1757 Virginia Tech, Blacksburg, Virginia 24061, United States; ‡ Center for Ecosystem Forecasting, Virginia Tech, Blacksburg, Virginia 24061, United States; § Department of Forest Resources and Environmental Conservation, Virginia Tech, Blacksburg, Virginia 24061, United States

**Keywords:** adaptive water management, ecological forecasting, explainable AI, machine learning, multi-horizon forecasting, phytoplankton, uncertainty quantification, SHAP (SHapley additive exPlanations)

## Abstract

Phytoplankton blooms threaten drinking-water security, yet translating high-frequency monitoring data into timely, reliable, and interpretable forecasts remains challenging. Machine learning (ML) models can improve predictive accuracy but typically require intensive tuning, yield deterministic forecasts without uncertainty, and offer limited model explainability. To address these limitations, we present here an end-to-end framework integrating an extreme gradient boosting (XGBoost) ML with automated hyperparameter optimization, bootstrap-ensemble uncertainty quantification, and interpretability through SHapley additive exPlanations (SHAP), which we applied to forecast phytoplankton in a water-supply reservoir. A 10-member ML ensemble achieved high chlorophyll-a forecast skill and uncertainty quantification, improving continuous ranked probability score (CRPS) by up to 14.6% over the single model, with ensemble CRPS of 1.2 and 2.7 μg/L at 1 and 7 days ahead, respectively. Global SHAP explanations identified initial chlorophyll-a as the primary driver of the forecast 1 day ahead, with thermal stratification and dissolved organic matter gaining prominence at 7 days. Local SHAP attribution showed individual events to be physically interpretable, each traceable to the dynamic influence of thermal stratification, meteorological forcing, and initial chlorophyll-a. This lightweight transferable framework advances mechanistic understanding of phytoplankton dynamics and supports proactive, risk-informed water-quality management.

## Graphical abstract

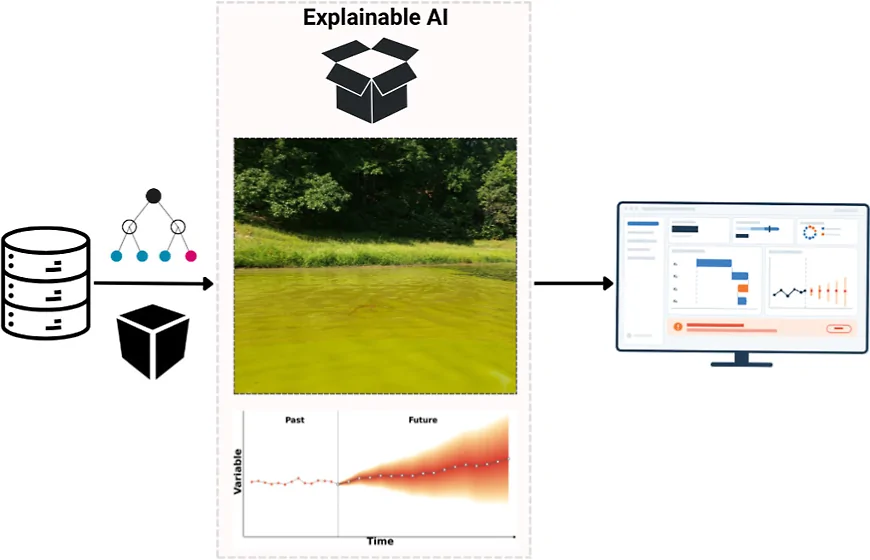

## 1 Introduction

Warming temperatures and intensifying anthropogenic stressors are increasing the frequency and severity of harmful phytoplankton blooms worldwide,^1–3^ motivating new approaches for their management. These blooms pose severe threats to drinking water safety due to their toxins, scums, and odors.^4^ In response to this need, water managers are seeking new tools for proactive and pre-emptive decision support, including forecasts.^5^ Ecosystem forecasts, i.e., predictions of future environmental conditions with specified uncertainty,^6^ have much promise for aiding bloom risk management, including providing anticipatory information to inform drinking-water advisories, recreational closures, and fisheries guidance.^7^ For maximum utility as decision support tools, these forecasts need to be accurate, near-real time, and automatically updated as new monitoring data arrives. They must also provide actionable insights and quantified uncertainty to communicate risk appropriately.^8^

Several different modeling approaches can be used for forecasting phytoplankton. For example, process-based mechanistic models offer physically interpretable predictions, but they are computationally expensive and difficult to operationalize because of complex boundary conditions, stringent numerical convergence requirements, and the need to calibrate many model parameters simultaneously.^9–11^ In parallel, machine learning (ML) modeling approaches have emerged as efficient alternatives for learning nonlinear relationships and capturing complex interactions.^12–16^ Moreover, the construction and deployment of ML models present fewer structural and parameterization challenges than their process-based numerical counterparts.^15^ Consequently, ML models are increasingly used to simulate and forecast phytoplankton blooms,^12,16,17^ from neural networks^18^ to more recent deep learning architectures.^19^

However, there are two main challenges that still need to be overcome before ML models can be widely employed for phytoplankton forecasting. First, ML models are “black box”, i.e., their predictions are not readily interpretable, hindering their usability by managers.^20^ While new explainable AI (XAI) tools are enabling modelers to begin to identify the relative importance of different features (model drivers) in ML models,^21^ their utility in an out-of-sample (future) forecasting framework remains underexplored. In particular, most applications of XAI to date have been confined to static post-hoc analyses,^22^ leaving the temporal evolution of forecast drivers across different horizons, or times into the future, largely untested. Second, uncertainty in ML predictions needs to be quantified to communicate bloom risk appropriately. Within water quality forecasting, most studies still provide deterministic point predictions, with comparatively limited adoption of probabilistic distributions to explicitly quantify uncertainty.^17^ Together, these challenges have limited the utility of ML models for probabilistic phytoplankton forecasting to date.

We propose to overcome these challenges and integrate XAI and uncertainty quantification into a near-term phytoplankton forecasting framework. First, XAI is beginning to open the black box nature of ML models, translating predictive accuracy into process-relevant insights. This “black box” barrier is especially consequential when forecasts are used by managers to trigger costly or disruptive interventions, requiring defensible model justification alongside predictive skill. In this regard, the SHapley additive exPlanations (SHAP) an XAI tool offers a pathway from predictions to decisions by decomposing model outputs into actionable driver contributions.^23^ The technique can attribute a given phytoplankton bloom-risk forecast model to specific physical and biogeochemical drivers (e.g., thermal structure, mixing conditions, nutrients), enabling scientific interpretation and validation.^24^ Critically, effective model explanation is not one-size-fits-all:^25^ the content and form of explanations must be aligned with the intended users and management needs, so that XAI-enabled interpretability can be actually usable for decision support, rather than just providing a generic feature ranking.

Second, quantification of predictive uncertainty is essential for deploying freshwater forecasting systems for decision support, as intrinsic stochasticity in aquatic ecosystems fundamentally limits the accuracy of predictive models.^26,27^ While ML adoption is rising in predictive applications, most ML models do not specify uncertainty and primarily use deterministic models and evaluation metrics.^17^ This gap persists even though uncertainty can be derived from multiple sources, including model (parameter) uncertainty, arising from limited knowledge and training data, and observational data uncertainty, arising from measurement noise and inherent variability in the data-generating process.^28,29^ Model and observational uncertainty can be referred to as epistemic (reducible with better data/modeling) and aleatoric (largely irreducible) uncertainty, respectively.^30,31^ Both forms of uncertainty contribute to total forecast uncertainty.

Ensemble methods are a well-established approach for robust uncertainty quantification (UQ) and risk assessment and are used extensively in weather forecasting.^32,33^ However, the operational deployment of such frameworks, particularly multi-model architectures, is often constrained by the computational demands of running a suite of physical simulations in real time.^34^ Consequently, ML models provide a distinct advantage as they are capable of not only capturing nonlinear dynamics but also generating forecasts in lower processing times, once models are trained.^35^ However, the application of ensemble methods remains sparse in ML-based water quality forecasting. In this context, the application of an ensemble ML modeling framework may offer a promising approach to quantify uncertainty and enhance model performance.

To address these gaps, we leveraged high-frequency, in situ water quality observations and automated hyperparameter optimization (HPO) to train an extreme gradient boosting (XGBoost) model to generate phytoplankton forecasts with associated explanations of the forecasted dynamics. Our overarching aim was to determine whether near-term phytoplankton forecasting can be made simultaneously skillful, uncertainty-aware, and interpretable. We addressed this aim through the following three objectives: (1) develop and evaluate a multi-horizon (one to seven day-ahead) ML model for forecasting phytoplankton, (2) quantify forecast uncertainty using a bootstrap ML model ensemble and assess how ensemble size influences probabilistic skill, and (3) attribute forecasts to their drivers with SHAP across horizons at both global (i.e., mean SHAP over all forecasts) and event scales (i.e., additive decomposition of an individual forecast). Our framework advances water quality forecasting by coupling predictive uncertainty with driver-resolved interpretability, thereby enabling more informed and actionable reservoir management decisions. Moreover, the proposed workflow is transferable to other systems and provides a blueprint for deploying ML models in real-time environmental forecasting applications.

## 2 Data and Methods

### 2.1 Study Site and Data Collection

We applied our framework to Beaverdam Reservoir (37.31°N, 79.81°W; BVR), a small drinking-water reservoir in southwestern Virginia, USA (Figure 1), managed as a secondary drinking water supply by the Western Virginia Water Authority (WVWA). BVR is a meso-eutrophic, dimictic reservoir that intermittently exhibits phytoplankton blooms.^36^ High-frequency in situ water quality measurements were collected from July 2020 through December 2024 at the reservoir’s deepest basin near the intake infrastructure using a YSI EXO2 (YSI Incorporated, a Xylem brand, Yellow Springs, Ohio, USA) multiparameter sonde deployed at approximately 1.5 m depth.^37^ The sonde recorded in situ water quality variables such as chlorophyll-a (Chl-*a*; a proxy for phytoplankton biomass), water temperature, and fluorescent dissolved organic matter (fDOM) at 10 min intervals, which were aggregated to daily means for analyses.

**Figure 1:**
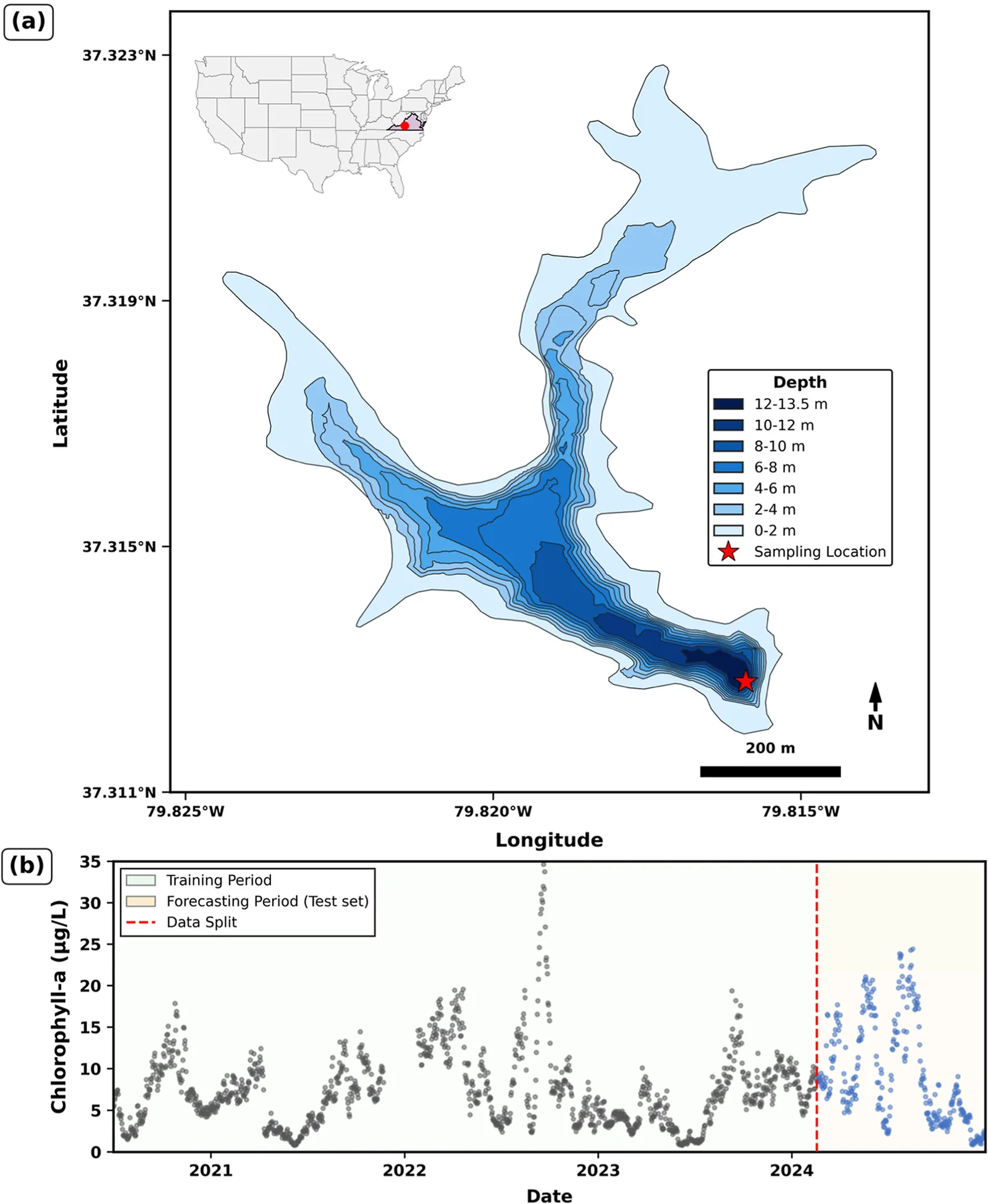
(a) Geographic location of Beaverdam Reservoir in Virginia, USA, and bathymetric map with depth contours. (b) Time series of high-frequency chlorophyll-a measured in Beaverdam Reservoir. The dashed vertical line denotes the chronological split between training and forecasting periods for out-of-sample model evaluation, with *n* = 1240 days in the training period and *n* = 311 days in the forecasting period.

To quantify the thermal structure and stratification controls on blooms, a vertical thermistor array (1 m spacing) extended from the near-surface to just above the sediment at the reservoir’s deepest site, enabling full water-column temperature profiles.^37^ In addition to in-reservoir observations, we calculated thermal stratification as Schmidt stability to evaluate lake thermal structure, as it plays a crucial role in controlling phytoplankton dynamics in lakes and reservoirs.^38^ We used the *Schmidt stability* function in the rLakeAnalyzer R package,^39^ after converting water temperatures to density and integrating stability over depth. Additionally, a dissolved oxygen sensor was deployed near the deepest thermistor (approximately 12 m) to monitor hypolimnetic oxygen conditions, which can control nutrient cycling and bloom persistence.^40^ Meteorological forcing was monitored using a nearby research-grade Campbell Scientific station located ∼2.1 km from the aquatic sensors, and included air temperature, wind speed, precipitation, and shortwave radiation.^41^ All data were obtained from published quality-controlled data products in the Environmental Data Initiative repository.^37,41^ For our analyses, we averaged data to a daily timestep except for precipitation, which was summed over the day and missing values were retained without imputation. Further definitions, units, ecological relevance, and summary statistics of each variable are provided in Supporting Information Table S1.

### 2.2 XGBoost Machine Learning Model

Figure 2 summarizes our workflow for one to seven day-ahead forecasting. We first developed and trained our ML model using historical data, then applied it for forecasting on out-of-sample data. Our workflow proceeded in four stages: (i) XGBoost model specification, (ii) automated hyperparameter tuning (iii), forecast uncertainty quantification using bootstrap ensembles, and (iv) post-hoc forecast explainability using SHAP to attribute predictions to input drivers across forecast horizons. The input features for our ML model included the current Chl-*a* concentration and a suite of biogeochemical and meteorological covariates described in Section 2.1, mapped directly to target Chl-*a* values for each specific forecast horizon. Although a few predictors show correlations with other predictors (Supporting Information Figure S1), gradient boosted ensemble models (i.e., XGBoost) remain robust and stable to multicollinearity.^42–44^ We, therefore, retained all the predictors, each capturing a distinct aspect of reservoir physics or water quality as applied in recent ML studies.^37,41^

**Figure 2:**
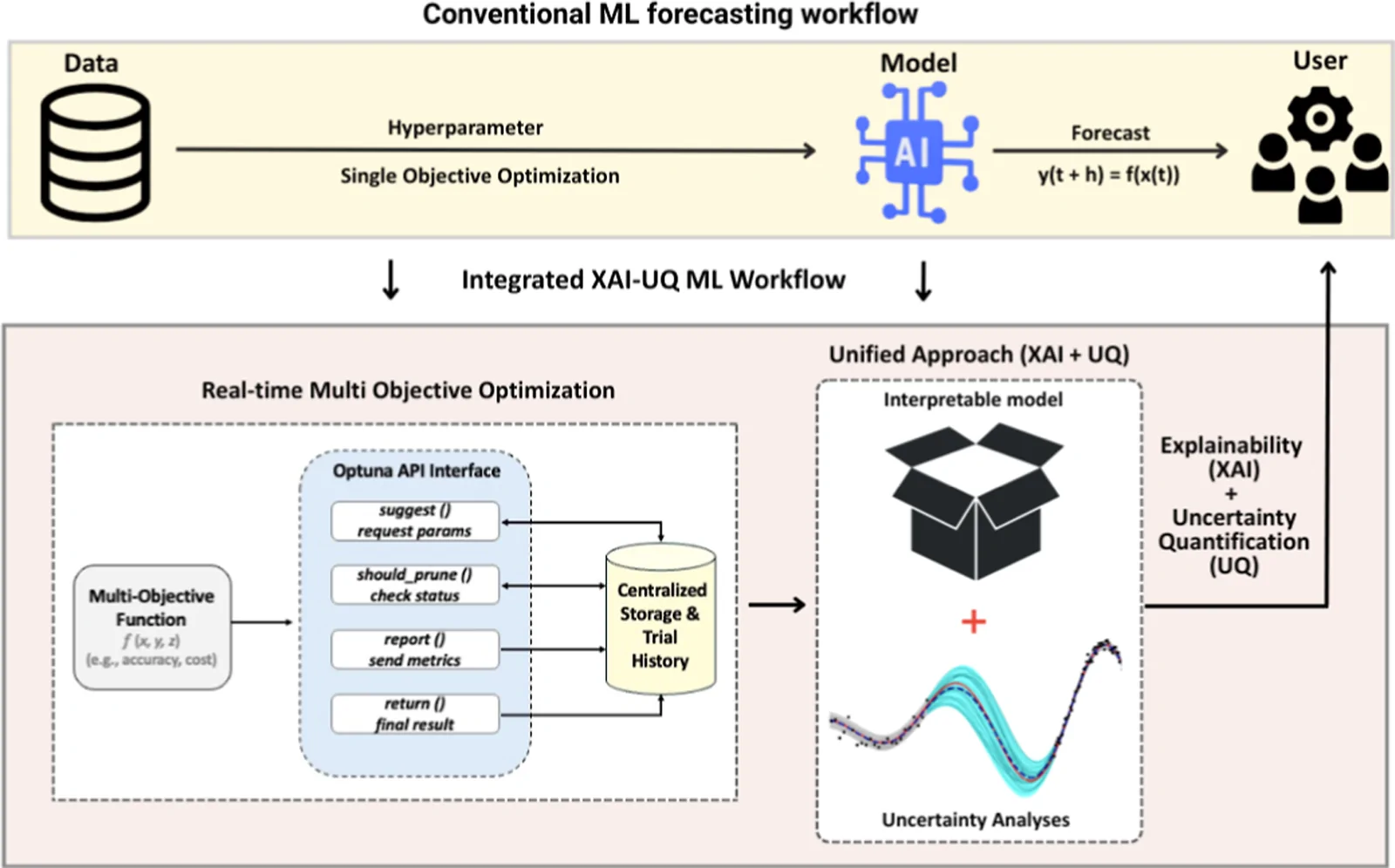
Overview of our conceptual framework, depicting a conventional ML workflow (top) and the integrated explainable AI and uncertainty quantification (XAI-UQ) framework (bottom) applied in this study.

Notably, we adopted a direct multi-step forecasting strategy rather than a recursive approach (Supporting Information Figure S2). Specifically, our forecasting model generated each day-ahead horizon’s prediction separately vs generating the next day’s forecast using the prior day’s forecast as an input. Consequently, we generated seven unique forecasts for 1 day-ahead, 2 days-ahead, etc. This approach mitigates error accumulation and prevents information leakage by conditioning forecasts solely on observed initial states at the day that the one- to seven-day-ahead forecast was initiated, rather than recursive predictions.

#### 2.2.1 Model Description

XGBoost is a scalable, regularized implementation of gradient boosting that builds an additive predictive model in a forward, stage-wise manner.^45^ It minimizes a regularized objective of the form:

1 $$L=\sum_{i=1}^{n}l\left(y_{i},{ŷ}_{i}\right)+\sum_{k=1}^{K}\Omega \left(f_{k}\right)$$

where $l\left(y_{i},{ŷ}_{i}\right)$ measures the prediction error and Ω­(*f*_*k*_) penalizes model complexity. The model applies a second-order Taylor expansion of the loss function, enabling efficient estimation of tree structure and leaf weights. The regularization term is defined as:

2 $$\Omega \left(f_{k}\right)=\gamma T_{k}+\frac{1}{2}\lambda \omega_{k}^{2}$$

with *T*_*k*_ denoting the number of leaves in tree *k* and ω_*k*_ the corresponding leaf weights. The parameters γ and λ penalize tree complexity (number of leaves) and the magnitude of leaf weights (L2 shrinkage), respectively, balancing model flexibility and generalization.^45^

XGBoost models deliver strong predictive performance on high-dimensional, relatively small environmental data due to its regularization, internal handling of missing values, and highly optimized split-finding and parallel execution.^45–47^ These properties are particularly valuable for phytoplankton forecasting, where system dynamics arise from nonlinear interactions among meteorological forcing, stratification, and nutrient inputs.^48^ Moreover, one review study^49^ revealed that XGBoost or a hybrid XGBoost-based model yielded the best performance (74%; 53 out of 72 studies) compared to other ML models in diverse water resources applications. Further, XGBoost integrates naturally with SHAP, enabling both local and global interpretability and supporting process-level understanding alongside prediction.^50^ Based on these advantages, XGBoost was selected as the forecasting model for this study and applied to generate near-term phytoplankton forecasts for one- to seven-days-ahead, balancing accuracy, efficiency, and explainability within a single forecasting framework. The model was trained on daily, high-frequency driver data, as detailed in Supporting Information Table S1, using an 80/20 chronological time-series split (Figure 1) to preserve temporal integrity and simulate realistic forecasting conditions. A separate model was trained for each lead time from one to 7 days using the Scikit-learn^51^ MultiOutputRegressor, mapping predictors at forecast issue time to chl-*a* at that horizon. Specifically, the model was trained on BVR data from 1 July 2020 to 8 February 2024, and the trained model was tested during the out-of-sample forecasting period of 9 February 2024 to 24 December 2024 as part of our reforecasting analysis. ML analyses were implemented in Python using the Scikit-learn package.^51^

#### 2.2.2 Hyperparameter Optimization

In this study, hyperparameter optimization was conducted using Optuna, an open-source framework designed for scalable and intelligent tuning of machine-learning models.^52^ Optuna implements a tree-structured parzen estimator (TPE) within a Bayesian optimization framework, enabling efficient navigation of high-dimensional hyperparameter spaces through adaptive sampling and early pruning of underperforming configurations.^52^ Additional capabilities include dynamic adjustment of search spaces, parallel and distributed execution, and integrated visualization tools to support both computational efficiency and transparent model development.

Optuna’s optimization workflow is structured around three core components (Figure 2): the objective function, the trial, and the storage.^52,53^ The objective function defines the performance metric and the associated hyperparameter domains. Each trial corresponds to the evaluation of a specific hyperparameter configuration, using five-fold time series cross-validation within the training partition, while the study orchestrates the overall optimization process by recording outcomes and guiding subsequent sampling decisions. This adaptive process drives convergence toward optimal configurations, ensuring the computational efficiency, scalability, and reproducibility requisite for data-driven forecasting.^54^ We ran 50 Optuna trials, and the resulting optimized hyperparameters used in this study are reported in Supporting Information Table S2.

#### 2.2.3 Uncertainty Quantification

Uncertainty analysis was an important aspect of our study aimed at improving the robustness and reliability of ML models for forecasting. We used a bootstrap-based ensemble to quantify uncertainty and improve our forecasting framework. Ensemble learning improves predictive performance by combining outputs from multiple models trained on different realizations of the data.^55–57^ In this study, an ensemble framework was constructed using bootstrap resampling. The ensemble size (*B*) was capped at *B* = 10, following a sensitivity analysis of *B*∈{2,3,5,7,10}, which revealed a threshold beyond which performance plateaued. In this approach, a set of *B* bootstrap samples was generated by drawing *n* = 1240 observations with replacement from the original training dataset, such that each resampled dataset (*B*_j_) is given by:

3 $$B_{j}=\left\{B_{1,j},B_{2,j},...,B_{N,j}\right\},j=1,...,B$$

Each bootstrap dataset contains approximately 63.2% unique observations (∼784 of 1240), with the remainder comprising duplicates, consistent with standard bootstrap sampling theory.^58^ Each bootstrap dataset, thus represents a compositionally distinct resampled version of the training data. A separate XGBoost model was trained on each bootstrap dataset, and the resulting suite of models formed an ensemble. Final predictions were obtained by aggregating individual model outputs, while the variability among ensemble members provided a basis for quantifying uncertainty.

#### 2.2.4 Model Evaluation

We used multiple metrics for evaluating model and forecast performance. Model accuracy was evaluated using root mean squared error (RMSE), computed between observed and predicted Chl-*a* for each forecast horizon. Probabilistic forecast skill was assessed using the continuous ranked probability score (CRPS). CRPS measures the squared difference, across all possible threshold values, between (1) the forecast cumulative distribution function (CDF) and (2) the step function representing the observed outcome.^59^ These differences are integrated over the full range of the variable, so lower CRPS values indicate a predictive distribution that is both closer to the observation and better balanced between sharpness and calibration.^60^ For bootstrap ensembles, CRPS was calculated from the empirical distribution of ensemble predictions. CRPS analyses provided an appropriate probabilistic assessment by enabling fair comparison across forecast horizons and against a deterministic model. We calculated these metrics for each horizon in each one- to seven-day-ahead forecast (*n* = 311) generated during the forecasting period (Figure 1).

#### 2.2.5 Explainable AI: SHAP

To interpret the trained XGBoost forecasting model and identify the dominant drivers of phytoplankton variability, we performed a post-hoc explainability analysis using SHAP across the one- to seven-day-ahead forecast horizon. This analysis was used to examine whether the factors contributing to the prediction changed with horizon. We summarized feature effects using SHAP beeswarm and illustrated case-specific behavior using SHAP waterfall plots. SHAP attributes each prediction to individual input features using game-theoretic Shapley values, assigning an additive contribution to every feature based on its average marginal effect across all possible feature subsets.^23^ Explanations are available at both global and local scales: global importance is obtained by aggregating SHAP values across all samples, while local interpretability is achieved by decomposing individual forecasts into feature-wise contributions.^50^ A key property is additivity, such that for each instance, the sum of SHAP values and the baseline prediction equals the model output.^23^ Positive SHAP values indicate that a feature increases the predicted Chl-*a* relative to the baseline, whereas negative SHAP values indicate a decreasing contribution.

## 3 Results

### 3.1 Evaluation of Forecast Horizon on Model Performance

The single XGBoost model demonstrated a robust predictive performance for short-term phytoplankton forecasting, with test (forecasting period) RMSE increasing monotonically from ∼2.1 μg/L for 1 day-ahead forecasts to ∼4.1 μg/L for seven day-ahead forecasts, when aggregated over the forecasting period (Figure 3a). As expected, predictive performance decreased as the forecast horizon extended, with the highest precision for a one-day-ahead forecast (train period RMSE ∼1.3 μg/L; test period ∼2.0 μg/L). Further, a stable train–test RMSE gap of ∼0.8 to ∼2.0 μg/L across the one-to-seven-day horizon confirmed consistent generalization to out-of-sample data. Across the one-to-seven day horizon, training mean bias error ranged from −0.001 to +0.013 μg/L (mean + 0.009 μg/L), less than 0.2% of the mean observed concentration, indicating negligible systematic bias in the training fit.

**Figure 3:**
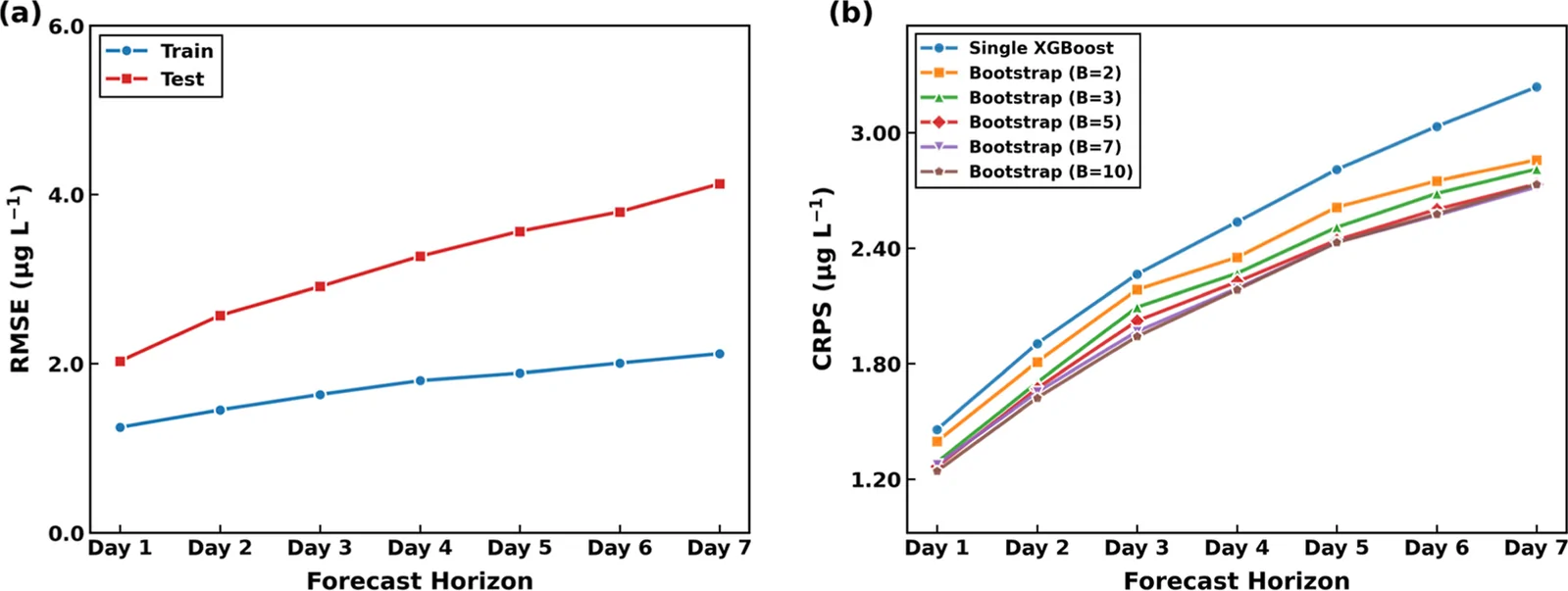
Evaluation of predictive accuracy and probabilistic skill across the one to seven day forecast horizon. (a) Evaluation of the single XGBoost model using root mean squared error (RMSE) aggregated across the training and testing (forecasting) periods. (b) Probabilistic performance of the single XGBoost model vs. bootstrap model ensembles with varying numbers (B) of ensemble members, assessed via the continuous ranked probability score (CRPS) aggregated over the forecasting period.

Correspondingly, probabilistic forecasting skill declined monotonically with forecast horizon, with single XGBoost CRPS increasing from ∼1.4 μg/L at 1 day-ahead to ∼3.1 μg/L at 7 days-ahead, reflecting the progressive expansion of forecast uncertainty with lead time (Figure 3b). The bootstrap ensemble consistently outperformed the single model across all horizons through variance reduction, with improvements in distributional accuracy becoming increasingly pronounced at longer lead times. Among the tested ensemble configurations, the *B* = 10 ensemble achieved the strongest probabilistic skill, with CRPS ranging from ∼1.2 μg/L at one-day ahead to ∼2.7 μg/L at seven-days ahead. Notably, ensemble gains were most pronounced at smaller sizes (*B* = 2–5) and saturated by *B* ≈ 7–10, beyond which marginal improvements became negligible (Figure 3b). This identified the 10-member ensemble as an optimal skill-convergence threshold for computationally efficient uncertainty quantification in this study.

### 3.2 Horizon Scale Dynamics

Feature importance analysis revealed a clear transition in predictive drivers over the forecast horizon (Figure 4). While past Chl-*a* remained the dominant predictor of future Chl-*a* at all horizons, thus exhibiting a strong influence of initial conditions, its relative contribution decayed from ∼70% (1 day-ahead) to ∼50% (7 days-ahead). With increasing horizon, the model adaptively shifted more importance toward environmental and physical covariates, specifically fDOM and Schmidt stability (SS). This transition reflects the decline in Chl-*a* autocorrelation with lead time and forecasts correspondingly place more weight on the environmental predictions.

**Figure 4:**
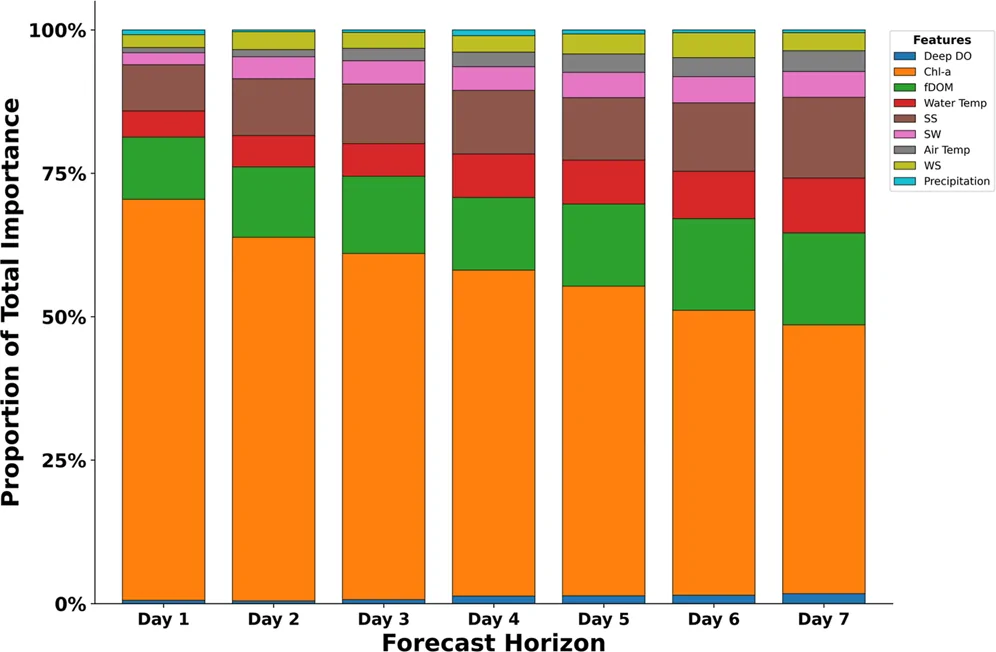
Proportional feature importance across the one- to seven-day forecast horizon, aggregated over the forecasting period. Here, Chl-*a* represents the model input initial Chl-*a*, fDOM is fluorescent dissolved organic matter, water temp is water temperature, SS is Schmidt stability, Deep DO is deep-layer dissolved oxygen, SW is shortwave radiation, air temp is air temperature, and WS is wind speed.

The SHAP beeswarm plot (Figure 5) provides a complementary, distribution-level view of how individual predictors influenced one- to seven-day forecasts aggregated across the dataset. Initial Chl-*a* remained the dominant feature one-week ahead, exhibiting the widest spread of SHAP values, confirming the strong influence of persistence even at this horizon. Among environmental drivers, fDOM and water temperature show predominantly positive, nonlinear contributions. In contrast, Schmidt stability exerted a largely suppressive influence, with higher stability values associated with negative SHAP contributions. Other meteorological variables contributed weakly and clustered near zero, indicating secondary or context-dependent effects (Figure 5).

**Figure 5:**
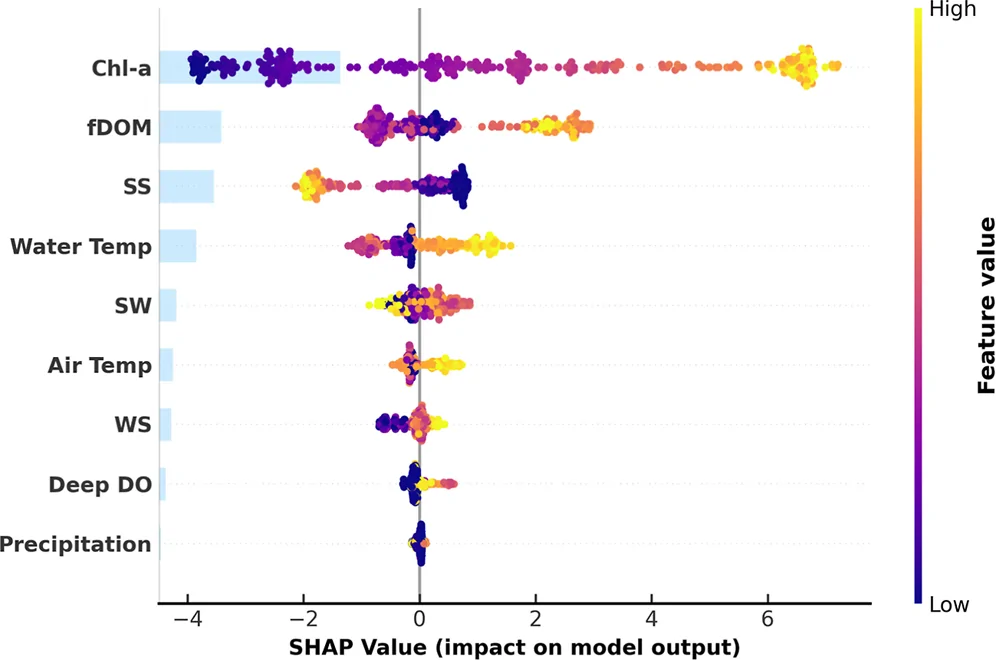
SHAP beeswarm plot showing the relative importance and directional influence of input features for the XGBoost model’s seven-day-ahead forecast, aggregated over the forecasting period. The light blue bars represent mean absolute SHAP values, indicating overall feature importance. The light blue bar length is proportional to the mean SHAP value and can be compared among bars but should not be interpreted using the SHAP values on the *x*-axis. Here, Chl-*a* represents the input initial Chl-*a*, fDOM is fluorescent dissolved organic matter, Water Temp is water temperature, SS is Schmidt stability, deep DO is deep-layer dissolved oxygen, SW is shortwave radiation, air temp is air temperature, and WS is wind speed.

### 3.3 Local Scale Interpretability

To advance our framework’s utility to explainable forecasting, we conducted horizon-scale attribution analyses using SHAP waterfall plots. This approach deconstructed individual seven-day forecasts across the forecasting period to isolate specific driver contributions and translate black box predictions into a decision support tool. As example use cases, we selected two dates exhibiting contrasting phytoplankton conditions (Figure 6): the first is a dynamically evolving bloom over the seven-day horizon starting on 4 June 2024, and the second is a sustained decline in phytoplankton over the seven-day horizon starting on 31 August 2024. These examples serve as a proof of concept for the value of local attribution, showing how the explainability framework can transform one- to seven-day forecasts into an actionable, driver-resolved decision support tool.

**Figure 6:**
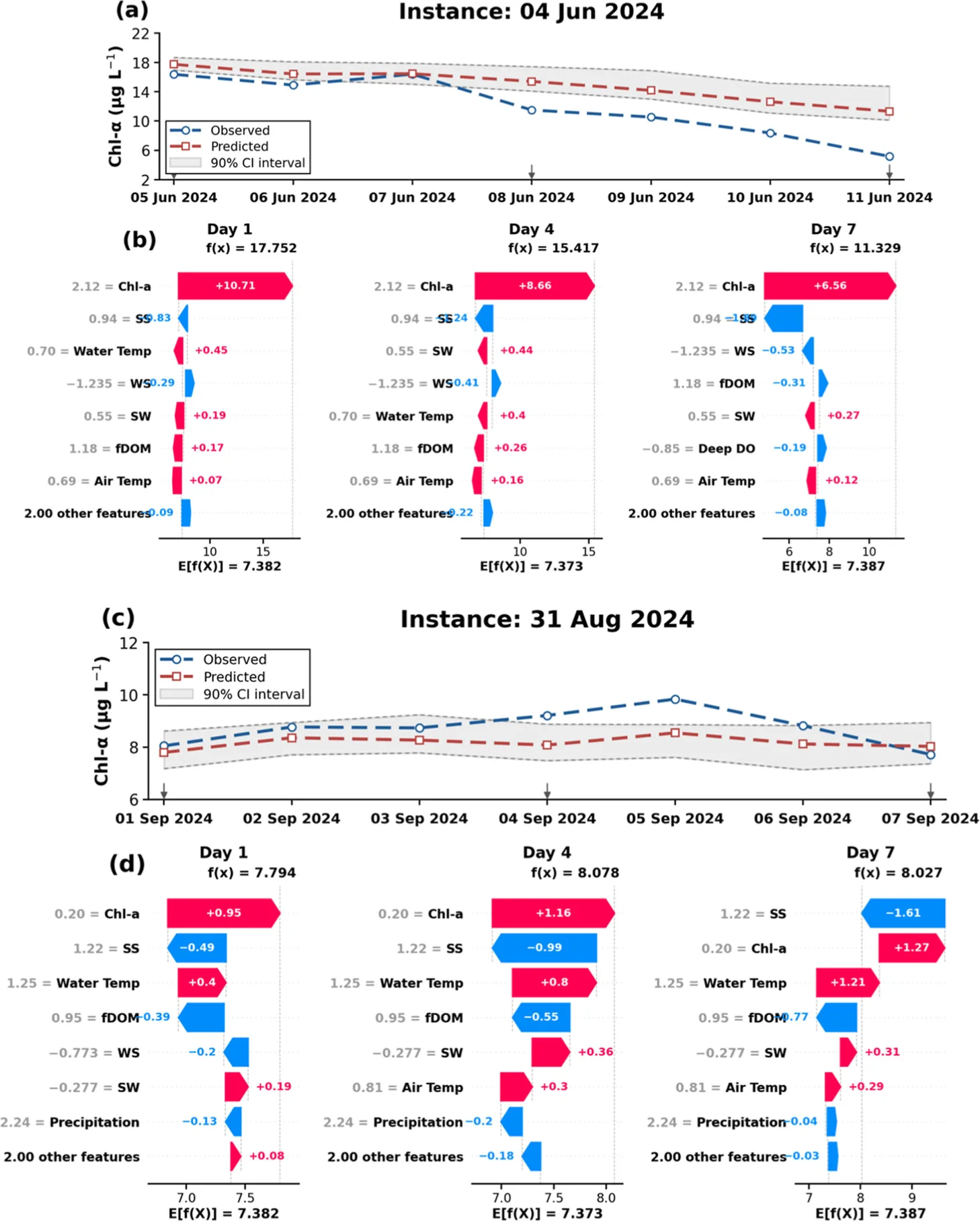
Observed versus predicted Chl-*a* concentrations and feature attribution for two forecasts generated on: (a,b) 4 June 2024 and (c,d) 31 August 2024. For each forecast, the top panels (a,c) show the seven-day horizon forecast and bottom panels and (b,d) present SHAP waterfall plots for day one, day four, and day seven in the forecast horizon. Red and blue bars denote positive and negative contributions, respectively, to the chlorophyll prediction.

For the first forecast starting on 4 June 2024 (Figure 6a), SHAP waterfall decomposition shows that this forecast was initially dominated by Chl-*a* but became progressively regulated by physical and biogeochemical controls with increasing forecast horizons. At 1 day-ahead, the prediction was driven primarily by initial Chl-*a* (+10.71 μg/L), indicating strong short-term persistence of antecedent biomass conditions. By 4 days ahead, this contribution had weakened (+8.66 μg/L), as radiative and thermal effects were increasingly offset by mixing-related suppression. By 7 days ahead, the persistence signal had declined further (+6.56 μg/L) and was counterbalanced by negative contributions from wind forcing, stratification, fDOM, and deep DO. This pattern indicates that, despite high initial biomass, environmental constraints limited further accumulation and led to stabilized or declining Chl-*a* trends.

For the second forecast starting on 31 August 2024 (Figure 6b), the forecast evolution reflected a more balanced combination of the initial system state and environmental drivers. On the one-day horizon, positive contributions from initial Chl-*a* (+0.95 μg/L) and water temperature (+0.40 μg/L) were offset by negative effects from Schmidt stability (−0.49 μg/L) and fDOM (−0.39 μg/L). At the four-day horizon, the contribution of initial Chl-*a* increased slightly (+1.16 μg/L), but stronger negative effects from Schmidt stability (−0.99 μg/L) and fDOM (−0.55 μg/L) pointed to increasing environmental regulation. At the seven-day horizon, initial Chl-*a* (+1.27 μg/L) and water temperature (+1.21 μg/L) continued to support higher Chl-*a*, but these effects were outweighed by stronger suppressive contributions from Schmidt stability (−1.61 μg/L) and fDOM (−0.77 μg/L).

## 4 Discussion

### 4.1 Overview

A key contribution of this study is advancing a water quality forecasting framework that supports a proactive, rather than reactive, approach toward phytoplankton bloom management. To date, bloom forecasting has been used primarily for advisories and to support reactive responses.^61^ Here, we present a multi-horizon XAI framework driven by high-frequency in situ data to generate one- to seven-day-ahead proactive Chl-*a* forecasts with uncertainty quantification. Critically, this framework makes forecasts more actionable by resolving drivers across multiple horizons, rather than relying on static, time-averaged feature importance. By applying SHAP attributions across one- to seven-day horizons, we uncover both local (day-specific) and horizon (week-specific) explanations. These analyses reveal changing controls from intrinsic biological persistence at one-day horizons to a greater role of extrinsic environmental forcing at one-week horizons, while local attributions resolve additional context-specific drivers. In parallel, we introduce a bootstrap-ensemble XGBoost approach that improves forecasts by reducing single-model variance and yielding calibrated, horizon-specific risk estimates. Together, these advances inform decision-relevant, risk-informed guidance that supports interventions to mitigate or avert bloom impacts (Figure 6).

Our forecasting framework provides skillful and uncertainty-informed Chl-*a* predictions across the one- to seven-day horizon, but the skill is strongly lead-time dependent. The highest skill was at short horizons and became progressively lower as the forecast window extended (Figure 3). This degradation in skill is expected and has been observed in other phytoplankton forecasting studies,^62,63^ including process-based models that generated forecasts of Chl-a over the same one-to-seven-day window as our study^64^ and a multi-forecast analysis that found Chl-*a* declined by up to 50% over the one- to seven-day ahead horizon.^65^ The degradation is likely due to forecasted drivers being less accurate further in the future, models missing key processes, and progressive loss of explanatory power from autoregressive processes that are strongly informed by recent observations.

### 4.2 Using a Direct Multi-Horizon Forecasting Approach

Our multi-horizon forecasting workflow uses a direct strategy (i.e., training one model for each forecast horizon in the one- to seven-day-ahead window) rather than a recursive/iterated strategy that repeatedly applies a one-step model and feeds predictions back as inputs to reach longer lead times. Recursive multi-step approaches are useful because they require fitting only a single model and generate a coherent forecast trajectory, but they are prone to error propagation: one-step biases can compound as predictions are recycled forward, degrading skill and inflating uncertainty with lead time.^66,67^

In our study, the direct forecasting strategy offers two practical advantages. First, it avoids explicit cross-horizon error accumulation from iterating predicted values. Second, it enables a horizon-specific mapping between available predictors and Chl-*a*, which is important when the dominant controls shift with the forecast horizon, as shown in our study. Crucially, this horizon-specific formulation also makes explainable feature attribution scientifically interpretable and actionable. Because each model is conditioned on observed predictors at the forecast issue time, the resulting attributions (e.g., SHAP values) can be interpreted as the marginal contribution of contemporaneous states and drivers to forecast skill at that specific horizon. The direct strategy might not be appropriate in all contexts, as fitting multiple models increases training and tuning effort. Also, at longer horizons, predictor–target linkages often become more diffuse, which can reduce forecast precision and the stability of inferred relationships.^68^ Overall, the choice of forecasting strategy should be application-dependent, but our analysis highlights the potential of the direct multi-horizon approach for phytoplankton forecasting.

While our results show promising near-term Chl-*a* forecast skill at BVR, performance can vary across different applications and is sensitive to site conditions, training data, available model predictor datasets, and optimization choices, as highlighted by the literature. For example, at nearby Falling Creek Reservoir, Woelmer et al.^62^ used linear autoregressive models to predict Chl-*a*, reaching an RMSE of 3.9 μg/L at 1 day-ahead and of 6.1 μg/L at 7 days-ahead. Lofton et al.^69^ compared 16 Chl-*a* models, including machine learning and process-based models, at the same reservoir as Woelmer et al.^62^ and found that regression models generally performed best across one- to ten-day horizons, with RMSE ranging from 2.6 to 6.9 μg/L. These studies were primarily focused on analyzing Chl-*a* forecast skill and did not include an analysis of the drivers explaining patterns in individual predictions, as done in this study. Beyond nearby waterbodies, data-driven research on algal prediction has been carried out across many sites worldwide, with our Chl-*a* forecast skill comparable to that reported for other systems. For example, one-to-seven-day-ahead RMSE for a hybrid ML-numerical model in Shanzai Reservoir, China,^70^ was 1.3 to 3.8 μg/L, the MAE for gradient boosting trees in western Lake Erie was 1.67 to 5.06 μg/L,^71^ and the one-week-ahead RMSE for a satellite-based statistical model in the Murray-Darling Basin, Australia, was 8.1 μg/L.^72^ However, differences in trophic status, data source, evaluation period, and model choice preclude direct comparison among studies. Broadly, our findings are consistent with prior work suggesting that boosting-based ensembles (i.e., XGBoost) often perform well in environmental applications by capturing nonlinear interactions in high-dimensional, noisy data with strong robustness and data efficiency.^49^ This is because XGBoost’s second-order (Newton-style) optimization and regularization curb overfitting and support strong generalization even with limited data.^45^ Finally, TPE-based Bayesian hyperparameter optimization provides an efficient route to high-performing configurations without exhaustive search, supporting scalable and reproducible forecasting deployment.^73^

Future work should focus on further evaluation of our forecasting approach across other monitored freshwater ecosystems. Our high-frequency data yielded a forecasting period that spanned a complete seasonal cycle and thus was sufficient to validate the framework. However, limited size of the overall dataset precluded robust season-stratified modeling. As more observational data are collected, future studies could develop season-resolved forecasting models, examine seasonal driver attribution, and evaluate more data-intensive models, including deep learning. Further, our forecast strategy fit the model once using the training data period, so the model calibration did not evolve as new data was collected; future applications could explore the influence of iterative model re-training on forecast skill and explainability.

### 4.3 XAI-Informed Variability in Drivers across the One- to Seven-Day Horizon

With increasing forecast horizon, SHAP attributions show a progressive decline in the influence of initial Chl-*a*, while slower-varying physical and environmental controls become relatively more informative (Figure 4). This shift reflects a transition from short-term biological persistence (“memory”) to increasing control by environmental drivers at longer horizons. Water temperature has both direct and indirect effects on phytoplankton. Directly, it sets phytoplankton metabolic and growth rates; indirectly, it controls the vertical density gradients and therefore the strength of thermal stratification, quantified here by Schmidt stability.^74^ In our system, phytoplankton blooms were most likely to occur during periods with weaker thermal stratification (indicated by low Schmidt stability), suggesting a mixing-driven mechanism in which weaker stratification enhances vertical exchange and episodic nutrient delivery from depth or sediments when light and temperature are favorable. This mechanism is seasonally modulated, as nutrients accumulate in bottom waters under summer anoxia and are released to surface water when stratification weakens.^75^ In parallel, fDOM integrates signals of internal biogeochemical processing, serving as a proxy for bioavailable organic nitrogen, carbon, and phosphorus that can alleviate nutrient limitation, stimulate photosynthesis, and, for some taxa, directly support growth via mixotrophy.^76–78^ Elevated fDOM can also modulate light availability and photochemical pathways, further shaping nutrient availability and microbial competition.^79,80^ Together, low Schmidt stability and elevated fDOM conditions capture slower-evolving physical–biogeochemical drivers that become increasingly predictive as lagged Chl-*a* loses influence at 1-week-ahead forecast horizons in our study system.

The differences in the most important model drivers of Chl-*a* among forecast horizons are consistent with broader evidence that Chl-*a* predictors are strongly system- and context-dependent. Prior studies have identified climatic and hydrological drivers such as air temperature, water level, and wind speed as key drivers in some systems,^81^ whereas in highly eutrophic lakes and reservoirs, nutrient supply and internal biogeochemical cycling can dominate bloom dynamics.^82^ Mixed results are common, with dominant drivers varying by season, trophic state, data coverage, and modeling choices.^83^ Collectively, these findings underscore that both Chl-*a* forecast skill and its driving features are site-dependent, motivating system-specific monitoring strategies.^24^ While our analysis used extensive high-frequency in situ water quality and meteorological observations, we acknowledge that other important variables, such as nutrients (phosphorus and nitrogen), were not included in our model because long-term, high-frequency measurements were not available for these drivers. Future work should incorporate these variables and others to better resolve biogeochemical processes and improve forecast skill, especially at longer horizons.

### 4.4 Uncertainty Quantification with a Bootstrap Ensemble

We scored the full predictive distribution against observations at each lead time so that forecast reliability is verified at the horizon on which drinking water managers act. Notably, only 26% (16 of 61) of recent near-term freshwater quality prediction papers included quantified uncertainty.^17^ Even when ensembles have been used to represent the uncertainty, forecast skill has been reported deterministically.^54,84^ Whether an ensemble improves probabilistic skill under a proper scoring rule therefore remains largely untested.

Our bootstrap ensemble consistently outperformed a single XGBoost model across all forecast horizons, with mean CRPS improving by up to 14.6% for the *B* = 10 ensemble. Performance gains plateaued beyond *B* ≈ 7 ensemble members, with <1% improvement observed between 7 and 10 members (Figure 3b). This saturation identifies a practical threshold at which further ensemble expansion yields negligible improvement, balancing computational cost against marginal gains in probabilistic skill. The ensemble’s advantage stems from variance reduction: whereas a single XGBoost configuration is susceptible to overfitting local noise, aggregating predictions across resampled subsets decorrelates errors and stabilizes forecast distributions. Notably, the residual spread beyond this plateau likely reflects limitations in the current predictor set and model structure rather than intrinsic bloom stochasticity, though our framework does not formally decompose these sources. This distinction matters in practice because quantifying the probability of exceeding phytoplankton bloom thresholds likely provides more actionable guidance for water managers than a single mean prediction. That said, the incremental nature of gains from a homogeneous ensemble may point to structural limitations of using a single model (i.e., XGBoost) across all ensemble members.^55^ Multi-architecture ensembles that combine structurally diverse models, such as long short-term memory (LSTMs) alongside gradient-boosted trees, may better capture complementary temporal and feature-based dependencies, warranting investigation in future work.^55^ It is also worth noting that inherent Chl-*a* variability within the training data may constrain bootstrap resampling diversity at larger ensemble sizes; however, systems with greater dynamic variability may yield stronger improvements from this ensemble approach. More broadly, following Harris et al.,^85^ we recommend that uncertainty quantification be adopted as standard practice in bloom forecasting to support risk-based decisions and transparent evaluation of probabilistic performance.

## 5 Conclusions

As phytoplankton blooms intensify under climate warming and eutrophication, their complex and nonlinear dynamics demand forecasting approaches that move beyond deterministic predictions toward actionable, risk-informed decision support with specified uncertainty. Addressing this need, our study demonstrates that multi-horizon phytoplankton forecasting can simultaneously deliver predictive skill, probabilistically calibrated uncertainty, and horizon-resolved explainability within a computationally accessible machine learning framework. Our results demonstrate that skillful near-term phytoplankton forecasting is attainable from in situ observations, with forecast performance remaining decision-relevant across the full one- to seven-day horizon.

Importantly, SHAP attribution revealed an ecologically interpretable shift in forecast drivers with increasing lead time: initial Chl-*a* dominated at short horizons, but its influence systematically declined to slower-varying physical and biogeochemical drivers as the forecast window extended to a week-ahead. Specifically, Schmidt stability and fDOM assumed greater explanatory weight at longer horizons, reflecting a transition from biological persistence to physical and biogeochemical forcing. Local waterfall attribution further translated these global patterns into event-specific driver diagnostics, demonstrating how the framework can move beyond black-box prediction to deliver horizon-specific insights for managers. Future work should incorporate high-frequency nutrient observations (e.g., phosphorus and nitrogen) to further deepen mechanistic insights, particularly at longer horizons when biogeochemical processes become increasingly dominant. The bootstrap ensemble consistently outperformed the single deterministic ML model in probabilistic skill across all horizons, with an optimal ensemble size of 10 members providing the best balance between uncertainty quantification quality and computational efficiency. Future extensions toward multi-architecture and/or multi-hyperparameter ensemble strategies^55^ offer a promising avenue to extend probabilistic skill beyond the limits of homogeneous ensembles. Finally, our framework’s lightweight, end-to-end design makes it well-suited for operational adoption and can be used to generate automated forecasts with associated horizon-specific explanations for informed decision making.

## Supplementary Material



## Data Availability

The datasets supporting this study are publicly accessible through the Environmental Data Initiative.^37,41^ All analytical code necessary to reproduce the findings is available on GitHub (https://github.com/rohitshukla01/BVR_Forecasting_Project), which will be archived and made openly available on Zenodo upon acceptance of the manuscript.

## Notes

Additional methodological details, including a correlation matrix of the variables used in the study, schematic of the forecasting strategy applied, summary statistics of the water quality drivers, and tuned XGBoost hyperparameters (PDF)
