# Supplementary material for: Advancing Near-Term Water Quality Forecasting with Explainable Machine Learning and Probabilistic Uncertainty Quantification
Source: ACS ES T Water. 2026 Sep 14;6(10):6601–12. doi: 10.1021/acsestwater.6c00757 (PMC13660380; doi:10.1021/acsestwater.6c00757)
Supplement: Supplementary file 1 [file ew6c00757_si_001.pdf]

Supplementary Information for:

Advancing Near-Term Water Quality Forecasting with Explainable Machine Learning and Probabilistic Uncertainty Quantification

Rohit Shukla[1,2*], Cayelan C. Carey[1,2], Adrienne Breef Pilz[1,2], R. Quinn Thomas[1,2,3]

[1]Department of Biological Sciences, Virginia Tech, Blacksburg, Virginia, USA

[2]Center for Ecosystem Forecasting, Virginia Tech, Blacksburg, Virginia, USA

[3]Department of Forest Resources and Environmental Conservation, Virginia Tech, Blacksburg, Virginia, USA

*Corresponding author: Rohit Shukla (rohitshukla@vt.edu)

<u>This file includes</u>:

Figure S1

Figure S2

Table S1

Table S2

Supplementary Figure 1: Pearson correlation coefficients (r) among the variables used in the study.

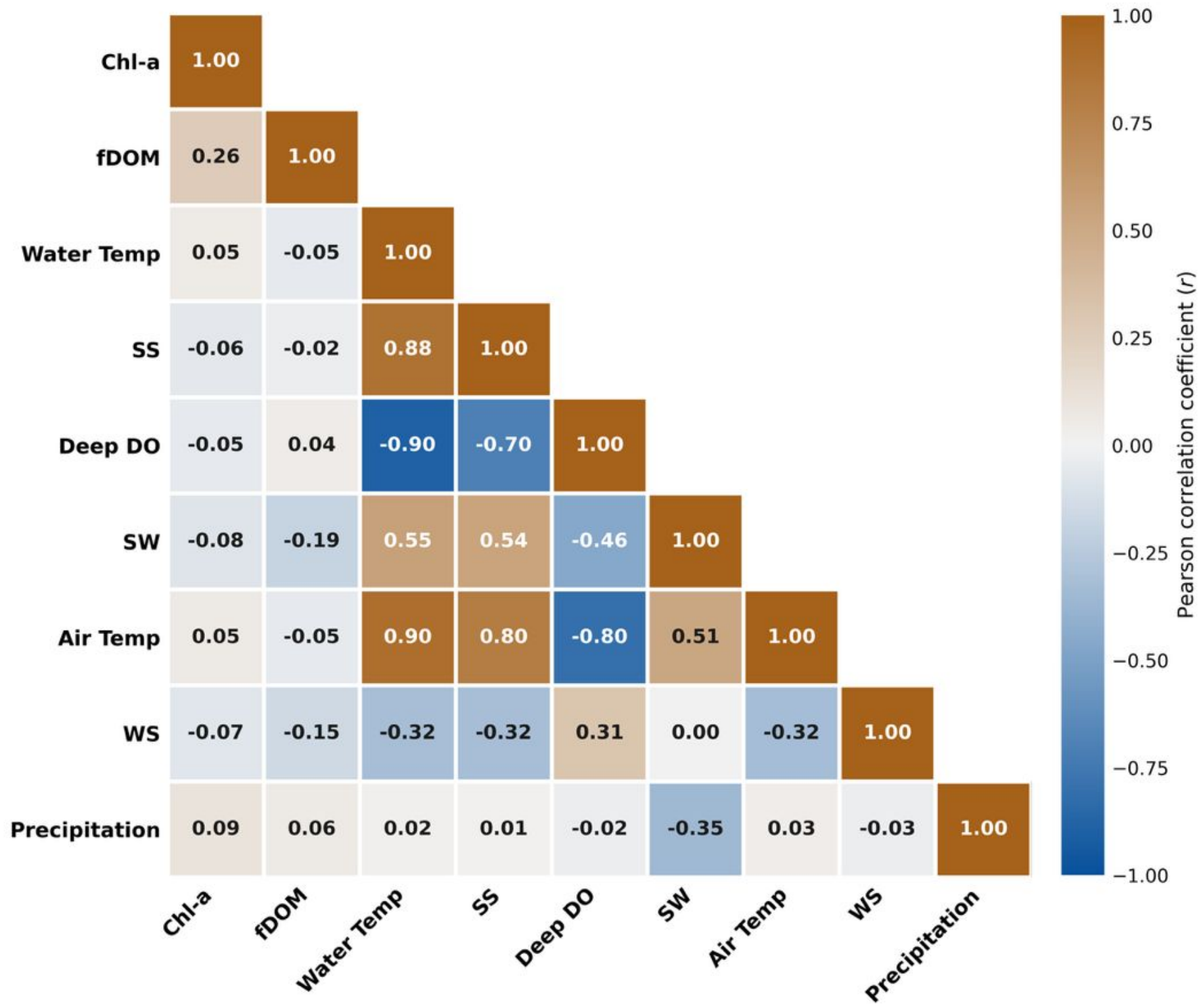

Supplementary Figure 2: Schematic of the direct multi-horizon forecasting framework used in this study. The x-axis represents time and the y-axis represents the observed variables (OBS) used to forecast an ecosystem variable on each day of a one to seven-day ahead forecast horizon. From each forecast origin date (i.e., the date a forecast is generated, denoted by the dark blue OBS circle), separate predictions are made individually for each horizon between one to seven days-ahead, as shown by the colored nodes. The grey bottom row shows the generic progression of the forecasting framework into the future.

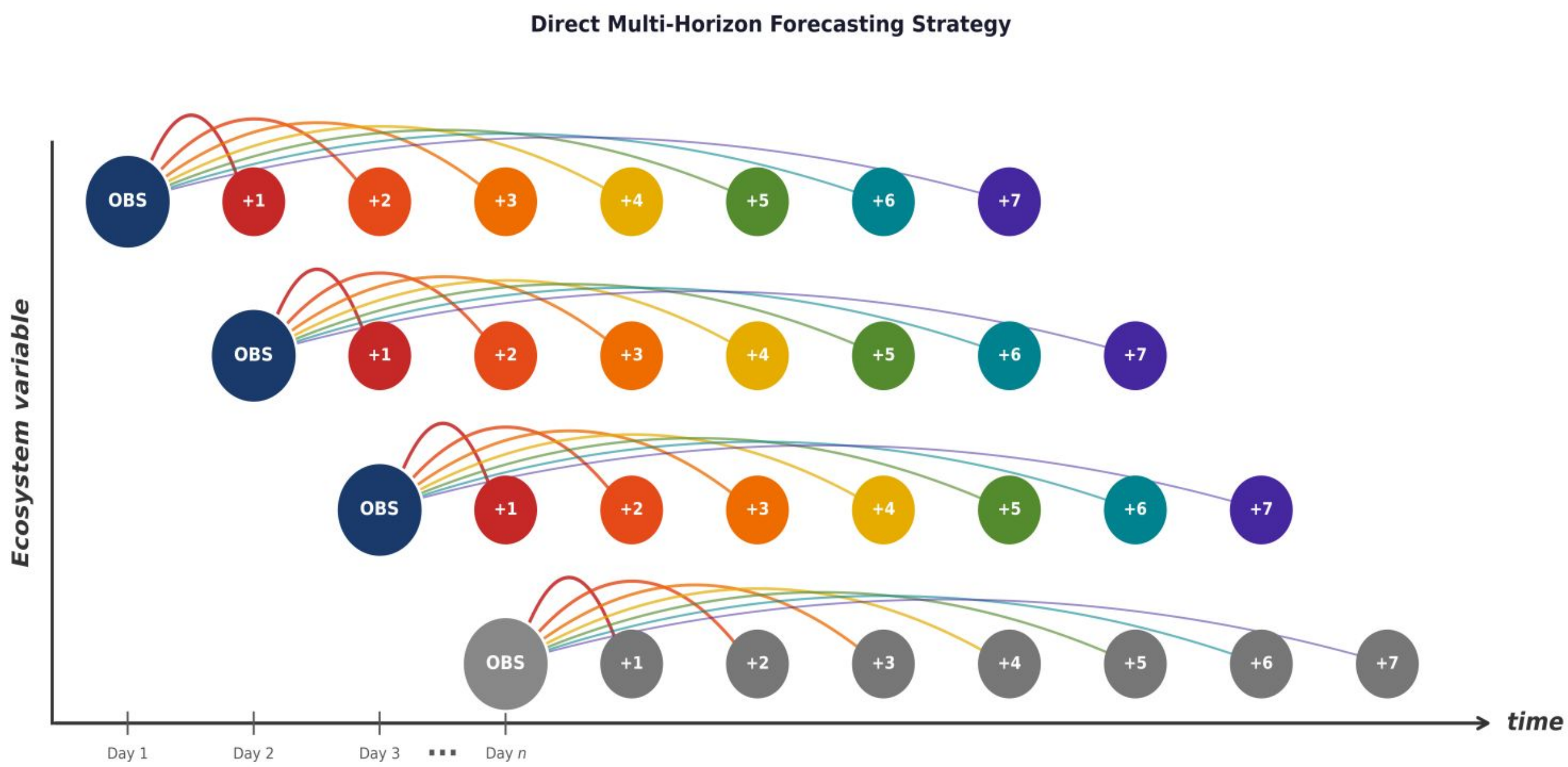

Supplementary Table 1. Summary of driver variables used for phytoplankton forecasting (Carey & Breef-Pilz 2025 a,b).

Variable with abbreviation and unit	Definition	Aggregation Frequency	Min	Max	Mean	SD
Chlorophyll-a (**Chl-***a*, μg/L)	Initial Chl-*a* concentration, which serves as a proxy for the autoregressive dynamics of phytoplankton	Daily mean	0.79	34.65	7.77	5.05
Water temperature (Water Temp, °C)	Thermal conditions in the water column at 1.6 m depth (surface layer)	Daily mean	0.28	28.87	16.77	8.48
Fluorescent dissolved organic matter (fDOM, QSU)	Proxy for dissolved organic matter concentration representing biogeochemical/optical effects	Daily mean	5.39	20.18	12.24	2.65
Schmidt stability, (SS, J m^{-2})	Metric of stratification strength in the full water column	Daily mean	-0.32	114.32	27.62	30.07
Dissolved oxygen (Deep DO, % saturation)	Concentration of hypolimnetic (bottom-layer) dissolved oxygen at the site, which serves as a proxy for redox-driven, internal nutrient cycling from the sediments into the water column	Daily mean	0.0	104.63	35.15	43.45
Air temperature (Air Temp, °C)	Air temperature	Daily mean	-12.58	28.49	15.12	8.19
Precipitation (mm)	Summed rainfall	Daily sum	0.0	78.49	2.82	7.53
Shortwave radiation (SW, W m^{-2})	Shortwave radiation available for phytoplankton growth	Daily mean	8.76	347.91	174.04	81.47
Wind speed (WS, m s^{-1})	Wind speed, which can alter physical mixing and bloom formation	Daily mean	0.06	7.87	1.88	0.75

Supplementary Table 2. Tuned hyperparameters used in the machine learning (XGBoost) model.

Hyperparameters	Optimized Values	Definition
n_estimators	487	Total number of boosting trees used in boosting
max_depth	6.00	Specifies the maximum depth allowed for each tree
learning_rate	0.023	Controls the contribution of new tree to the overall model
subsample	0.70	Represents the fraction of training observations sampled for each tree
gamma	1.39	Determines the minimum loss reduction required for an additional split
reg_alpha	9.66	Regularization term
reg_lambda	7.60	Regularization term
colsample_bytree	0.79	Denotes the fraction of predictor variables sampled when building each tree
